# Ancient Plumage Colour Genetics Reveal Goose Domestication and Hybridization

**DOI:** 10.1002/age.70165

**Published:** 2026-07-12

**Authors:** Johanna Honka, Suvi Olli, Arthur O. Askeyev, Igor V. Askeyev, Oleg V. Askeyev, Jouni Aspi, Dilyara N. Shaymuratova, Laura Kvist

**Affiliations:** ^1^ Ecology and Genetics Research Unit University of Oulu Oulu Finland; ^2^ The Institute of Problems in Ecology and Mineral Wealth Tatarstan Academy of Sciences Kazan Russia

**Keywords:** *Anser anser*, *Anser cygnoid*, *Anser fabalis*, *EDNRB2*, *MLANA*, plumage colour

## Abstract

In archaeological assemblages, differentiating the European domestic goose and its wild progenitor, the greylag goose ( *Anser anser* ), has been challenging due to their similar skeletal morphologies. A short region of mitochondrial DNA (mtDNA) analysed from ancient DNA (aDNA) has been shown to distinguish wild greylags from domestic geese, but some ancient specimens display atypical haplotypes or haplotypes belonging to other wild *Anser* species. Plumage colouration could verify the domestic status of archaeological geese, as white or mottled plumage would be indicative of domestication. We analysed a SNP upstream of the *EDNRB2* gene (*endothelin receptor B‐like*) linked to white spotting and a 1‐bp deletion in the Z‐chromosomal *MLANA* gene (*melan‐A*), causative of sex‐linked dilution colour (almost white male and light grey female) in European domestic geese. Together, white spotting and sex‐linked dilution result in white plumage. We also analysed a Chinese domestic goose (*A. cygnoid*)‐specific 14‐bp insertion within the *EDNRB2* gene, which also causes white colour, and searched for possible evidence of historical interbreeding between European and Chinese domestic geese. We detected several plumage colour patterns such as solid wild type, white spotted (i.e., saddleback), nearly white with some grey spots, and white or autosexing with of ‘almost white male and saddleback female’. Further, we discovered that European domestic geese had introgression from the Chinese domestic goose at least occasionally, from the 16th‐17th century Common Era, due to the presence of both European and Chinese domestic goose white alleles in a single individual.

When and where goose domestication occurred still remains uncertain, as does the evolution of geese since domestication (Honka et al. 2026). Geese were domesticated independently from two species: the European domestic goose from the greylag goose ( *Anser anser* ) and the Chinese domestic goose from the swan goose (*A. cygnoid*). Several modern European breeds show considerable ancestry (> 10%) from Chinese domestic geese (Heikkinen et al. 2020). This indicates that interspecific hybridization between the two domesticated lineages has been substantial. However, the exact timing of this hybridization remains unknown. It is assumed to be relatively recent and likely linked to breed development and human‐mediated crossings rather than ancient domestication events (Heikkinen et al. 2020).

Goose bones are often found in archaeological excavations, but their analysis is hindered by similar skeletal morphology within the genus *Anser*, making morphological identification especially challenging between domestic geese and their wild form (Albarella 2005; Mannermaa 2014). Additionally, geometric morphometrics, ZooMS (zooarchaeology by mass spectrometry), and dietary analyses with stable carbon and nitrogen isotopes have also been unsuccessful in differentiating domestic from the wild (reviewed in Honka et al. 2026).

Ancient DNA (aDNA) is a promising tool for distinguishing domestic and wild geese. A short fragment of a hypervariable portion of the mitochondrial (mtDNA) control region has been shown to be useful in identifying domestic geese (Barnes et al. 2000; Honka et al. 2018). However, mtDNA has some limitations as a few ancient geese were found to have mtDNA haplotypes that could not be assigned to domestic or wild forms (Honka et al. 2018). Additionally, some bones morphologically identified as domestic geese were genetically taiga bean geese ( *A. fabalis fabalis* ), raising questions about their identity (Honka et al. 2018). aDNA analysis of mutations underlying plumage colour patterns could reveal domestic geese (Mannermaa 2014), as leucistic geese are very rare in the wild and, thus, white or mottled geese would be definitive signs of a domestic population. To date, aDNA has not been applied to explore plumage colour in birds.

White plumage colour in the European domestic goose is caused by the action of two loci: one causing white spotting and the other a sex‐linked dilution, which in combination produces white (Jerome 1953). A 1‐bp deletion in exon 4 of the Z chromosomal *MLANA* (*melan‐A*) gene (NW_013185876.1: g.950868 C > −) is in perfect association with the sex‐linked dilution colouration in European domestic geese across different breeds (Yang et al. 2022; Olli et al. 2026), and thus can be confidently considered to be the causative mutation. The 1‐bp deletion causes a frameshift mutation leading to a change in the last 19 amino acids (Yang et al. 2022). The mutation is predicted to result in a longer protein product and abnormal function of the protein's C‐terminus (Yang et al. 2022). MLANA has a key role in melanosome development, and in pigeons (*Columba livia domestica*), copy number variants in the *MLANA* gene cause the Almond phenotype (variegated plumage colour; Bruders et al. 2020). The mutation causing spotting is yet to be identified, although a mutation upstream of the *EDNRB2* (*endothelin receptor B‐like*)/*LOC106047519* gene (NW_013185915.1: g.775151 G > T) is linked to solid colour in grey European domestic goose breeds (Yang et al. 2022, Olli et al. 2026). This genetic variant is located several tens of thousands of nucleotides upstream of the transcription start site and could result in downregulation of gene expression, and was identified as a strong candidate mutation for white spotting in domestic geese (Yang et al. 2022). Further, mutations in this gene cause spotted/piebald colouration across multiple avian species (Miwa et al. 2007; Kinoshita et al. 2014; Maclary et al. 2021, 2023; Xi et al. 2021; Nannan et al. 2024; Wang et al. 2025), see also Maclary and Shapiro (2025). EDNRB2 has a central role in the migration of melanoblasts and postmigratory melanocytes. Further genotyping of the mutation in *EDNRB2* (NW_013185915.1: g.775151 G > T) in multiple European domestic goose breeds revealed that guanine is strongly linked to white spotting and thymine to solid colour in European domestic goose breeds except in the Brecon Buff breed, while wild geese and Chinese domestic geese possess mainly guanine (Olli et al. 2026). Hence, the causative mutation for spotting in European domestic geese is yet to be discovered, but the guanine in g.775151 (NW_013185915.1) is indicative of white spotting in the European domestic goose background.

Geese homozygous only for the spotting allele show a saddleback pattern, and geese homozygous only for the sex‐linked dilution allele show autosexing (plumage colour difference between sexes) of the type ‘almost white male and light grey female’ as ZZ males are “double diluted” compared to ZW females (Olli et al. 2026). Other plumage colour patterns exist (autosexing of the type ‘almost white male and saddleback female’, buff and blue), but their genetic basis is unknown (Olli et al. 2026). In the Chinese domestic goose, white colour is caused by a codominant 14‐bp insertion in exon 3 of the *EDNRB2* gene (NW_013185915.1: g.750748–750 735 insertion; Xi et al. 2020, Yang et al. 2022, Ouyang et al. 2022, Yang et al. 2024, Olli et al. 2026). This insertion has been identified in multiple studies and across different Chinese domestic goose breeds. Hence, we are confident that it is the causative variant. However, plumage colour can be polygenic, as the *KIT* gene (*KIT proto‐oncogene, receptor tyrosine kinase*) has also been identified as a candidate gene for white plumage in Chinese domestic geese (Wen et al. 2021).

We aimed to investigate whether archaeological domestic geese could be identified through analysis of the sex‐linked dilution allele and the allele linked to spotting, i.e., by their plumage colour. As a case study, we used previously extracted aDNA from Medieval and Post‐Medieval (4th—18th century CE [Common Era]) archaeological geese (Honka et al. 2018). These specimens were identified as European domestic geese, putative European domestic geese/greylag geese and taiga bean geese by sequencing a short region of mitochondrial DNA (*n* = 51; Figure 1) as described in Honka et al. (2018). This previous aDNA extraction was performed using a silica spin‐column‐based method (Yang et al. 1998, as modified in Gamba et al. 2014 and Gamba et al. 2016), with slight modifications described in Honka et al. (2018). Here, we used samples that were previously successfully sequenced for an mtDNA fragment, and thus were proven to contain preserved aDNA (Honka et al. 2018). Authenticity of the aDNA was ensured by performing DNA extractions and PCR reaction setup in dedicated aDNA clean room facilities at the Centre for Material Analysis, University of Oulu, physically separated from other DNA and post‐PCR facilities. PCR reactions were pipetted in a UV‐sterilizing PCR workstation (Peqlab). Negative controls were used both in the DNA extraction and PCR stages. Filtered tips were used at all stages, and surfaces and pipettes were cleaned with DNA‐removing DNA/RNA‐ExitusPlus (ITW reagents) solution.

**FIGURE 1:**
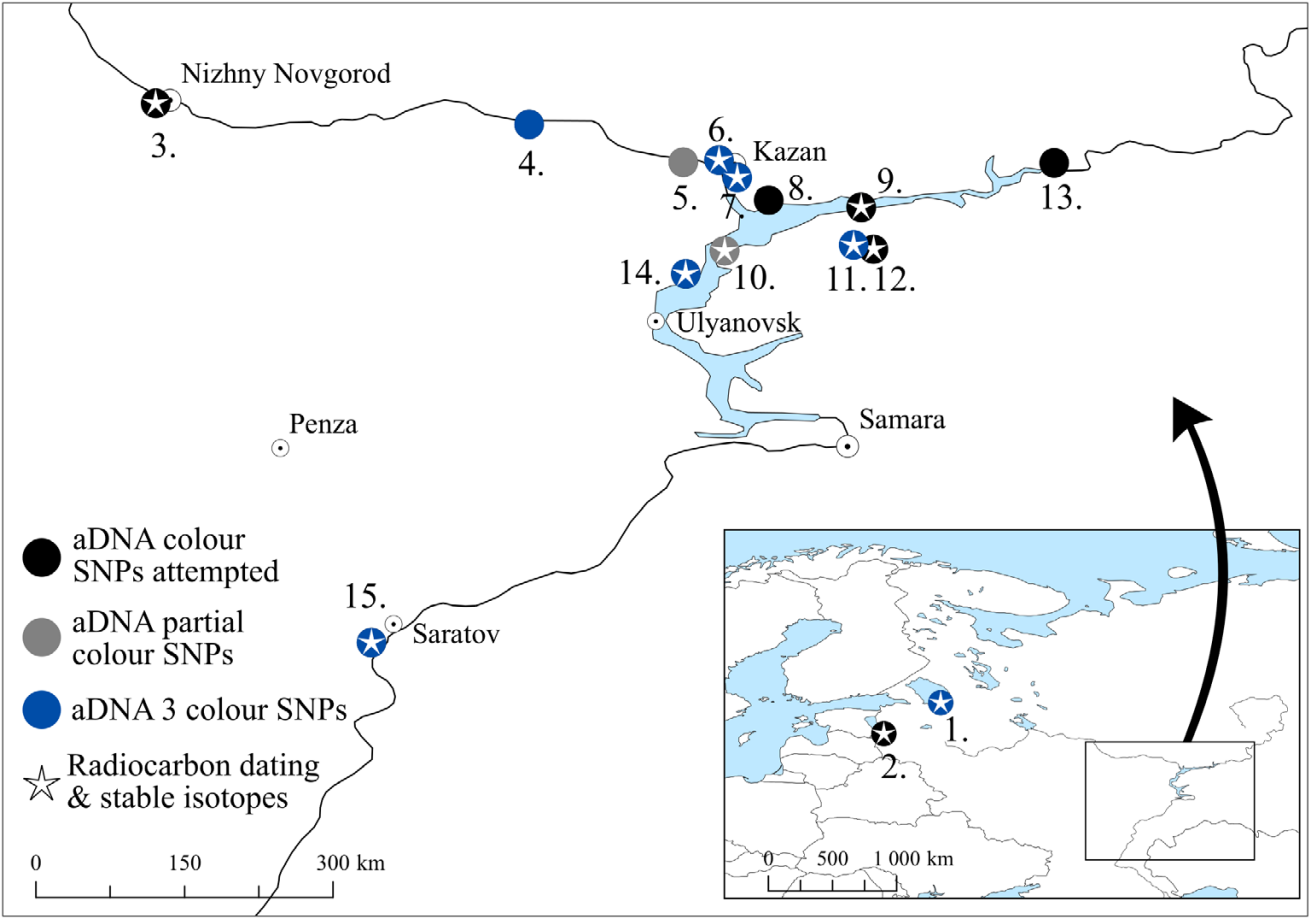
Locations of subfossil archaeological geese from the 4th to 18th century CE attempted for ancient DNA analyses of plumage colour polymorphisms, shown as black dots; partially amplified (1–2 loci) samples as grey dots; and successfully analysed samples for three loci as blue dots. White stars indicate locations with radiocarbon dating and carbon (δ^13^C) and nitrogen (δ^15^N) stable isotope measurements (Honka et al. 2026). 1. Staraya Ladoga (Leningrad Region), 2. Pskov city (Pskov Region), 3. Nizhny Novgorod Kremlin (Nizhny Novgorod Region), 4. Chebosakry city (Chuvash Republic), 5. Sviyazhsk (Tatarstan Republic), 6. Kazan Kremlin (Tatarstan Republic), 7. Kazan State University, Kazan city (Tatarstan Republic), 8. Imenkov hillfort (Tatarstan Republic), 9. Ostolopovskoe settlement (Tatarstan Republic), 10. Bulgar (Tatarstan Republic), 11. Toretskoe settlement (Tatarstan Republic), 12. Bilyarsk (Tatarstan Republic), 13. Elabuga hillfort (Tatarstan Republic), 14. Tetyushkoe II hillfort (Tatarstan Republic), and 15. Bagaevskoe settlement (Saratov Region).

We used two primer pairs, EDNRB2‐F/R and MLANA‐F/R (Olli et al. 2026), to genotype the SNP upstream of the *EDNRB2* gene and the 1‐bp deletion in the *MLANA* gene, respectively. No Chinese domestic goose bones or mtDNA were identified in the archaeological material (Honka et al. 2018), but we investigated the presence of the 14‐bp *EDNRB2* insertion in order to screen for evidence of crossbreeding background, using primer pair EDNRB2_indel‐F/R (Olli et al. 2026). Even though bone morphology and mtDNA did not indicate Chinese domestic goose ancestry, mtDNA is non‐recombining and maternally inherited and thus might not reveal genetic inheritance from the Chinese domestic lineage. All primers were designed based on the Chinese domestic goose whole‐genome sequence (NW_013185915.1) as described in Olli et al. (2026), as no annotated genomes of the greylag goose existed at the time. The designed primers were experimentally tested with both domestic goose types and greylag geese in Olli et al. (2026).

PCR reactions were performed in 12.5 μL volumes using 1× PCR buffer (HotStarTaq, Qiagen), 0.2 mM of each dNTP, 0.2 μM of MLANA‐F‐ and R‐primers, 2.5 mM MgCl_2_, 1 mg/mL BSA (bovine serum albumin), 4 U/reaction of HotStarTaq DNA Polymerase (Qiagen), and 1 μL of template DNA. PCR reaction setup was performed in the aDNA clean room facilities with negative controls. The thermocycling conditions were 95°C for 15 min, followed by 55 cycles of 94°C for 30 s, 60°C for 30 s, and 72°C for 30 s with a final extension of 72°C for 10 min. Results were visualized on 2% agarose gel, and successfully amplified samples were sequenced using the F‐primer with BigDye Terminator v3.1 (Applied Biosystems) chemistry. The reactions were run on an ABI 3730 sequencer (Applied Biosystems). We used CodonCode Aligner v4.0.4. (CodonCode Corporation) to manually edit the sequences and record the alleles. PCR reactions with either EDNRB2‐F/R or EDNRB2_indel‐F/R primers were performed similarly, but only for samples that were successful in PCR using the MLANA‐F/R (*n* = 18 out of the 51 attempted samples, Table S1), except that the annealing temperature was 63°C. Sequencing was performed similarly with the F‐primers. The 18 samples were sequenced twice or thrice. Six samples showed variable sequencing success between loci, and no phenotype was inferred from these samples (Tables 1 and S1). In an additional six samples, one of the loci failed to be sequenced twice despite multiple attempts, but a phenotype was inferred for these samples (Tables 1 and S1). We observed probable postmortem changes or polymerase errors in samples JH1 and JH62 (Tables 1 and S1). Phenotypic inference was based on Olli et al. (2026).

**TABLE 1:** Samples for which three mutations affecting plumage colour or linked to plumage colour were attempted for genotyping, their mitochondrial DNA sequencing results from a previous study (Honka et al. 2018), predicted phenotype and predicted domestic or wild status based on colour genotyping. Dating of the samples (based on archaeological stratigraphy or radiocarbon dating) and their location are also shown. Shading indicates samples for which all three loci were successfully sampled twice, and genotypes in parentheses indicate that the genotyping was successful only once out of two or three attempts. Due to sex‐linkage in the *MLANA* gene, it was impossible to differentiate homozygous males and hemizygous females, and thus we only show one allele (unless a heterozygous male) with C (cytosine) = no dilution, i.e., wild‐type and− 1 bp = sex‐linked dilution. In the locus upstream of the *EDNRB2* gene T (thymine) = linked to solid colour, i.e., wild type in European domestic goose and G (guanine) = linked to spotting in European domestic goose. In exon 3 of the *EDNRB2,*− = no insertion, i.e., wild‐type and 14‐bp insertion = Chinese domestic goose white.

Sample	Species based on mtDNA	mtDNA haplotype	Phenotype	Status based on phenotype	exon 4 in *MLANA*	upstream of *EDNRB2*	14 bp‐insertion in exon 3 of *EDNRB2*	Dating†	Site
JH24	*A. f. fabalis*	Fa5	Wild type	?	C	(T/G)^§^	−/−	4th–8th centuries CE	Tetyushskoe II hillfort, Tatarstan Republic
JH1	Domestic/wild greylag goose	F11	Wild type	?	C (C/T)*	G/G	−/−	7th century cal CE	Tetyushskoe II hillfort, Tatarstan Republic
JH40	*A. f. fabalis*	Fa3	Wild type	?	C	G/G	−/−	9th–11th centuries cal CE	Staraya Ladoga, Leningrag region
JH52	*A. f. fabalis*	Fa3			(C)		(−/−)	10th–12th centuries cal CE	Bulgar, Tatarstan Republic
JH35	Domestic/wild greylag goose	F6	(Wild type)		(C)	(T/T)		13th–14th centuries cal CE	Toretskoe settlement, Tatarstan Republic
JH28	Domestic	D4/D5	Saddleback	Domestic	C	G/G	−/−	13th–14th centuries CE	Bagaevskoe settlement, Saratov region
JH34	Domestic	D3/D7	(Saddleback)	Domestic	(C)	(G/G)	(−/−)	13th–14th centuries cal CE	Toretskoe settlement, Tatarstan Republic
JH33	Domestic	D3/D7		Domestic/wild	(C)			15th century CE	Toretskoe settlement, Tatarstan Republic
JH2	Domestic	D4/D5	Almost white male with grey spots	Domestic	C/−1 bp	G/G	−/−	16th–17th centuries CE	Kazan city, territory of Kazan State University, Tatarstan Republic
JH4	Domestic	D4/D5	(Saddleback)	Domestic	(C)	(G/G)	(−/−)	16th–17th centuries CE	Kazan city, territory of Kazan State University, Tatarstan Republic
JH8	Domestic	D3/D7		Domestic	(−1 bp)			16th–17th centuries CE	Kazan city, territory of Kazan State University, Tatarstan Republic
JH13	Domestic	D4/D5		Domestic	−1 bp			16th–17th centuries CE	Kazan city, territory of Kazan State University, Tatarstan Republic
JH17	Domestic	D4/D5	White and Chinese domestic goose white	Domestic	−1 bp	G/G	(14 bp insertion/14 bp insertion)^§^	16th–17th centuries CE	Kazan city, territory of Kazan State University, Tatarstan Republic
JH18	Domestic	D4/D5	White or autosexing white male saddleback female	Domestic	−1 bp	G/G	−/−	16th–17th centuries CE	Kazan city, territory of Kazan State University, Tatarstan Republic
JH61	Domestic	D3/D7	Saddleback	Domestic	C	G/G	−/−	16th–18th centuries CE	Cheboksary city, Chuvash Republic
JH62	Domestic	D3/D7		Domestic	−1 bp	(A/A)*, ^§^	−/−	16th–18th centuries CE	Cheboksary city, Chuvash Republic
JH65	Domestic	D4/D5		Domestic	(−1 bp)			17th century CE	Sviyazhsk, Tatarstan Republic
JH59	Domestic	D4/D5	(White or autosexing white male saddleback female)	Domestic	(−1 bp)	(G/G)	(−/−)	18th century CE	Kazan Kremlin, Tatarstan Republic

* Postmortem changes.

^†^ Dates reported as cal CE are based on a 95.4% probability from calibration of radiocarbon dates in Honka et al. (2026); otherwise, the dates are based on stratigraphy.

^§^ The sample was attempted thrice, but was successfully amplified only once.

Genotype G/G upstream of the *EDNRB2* gene is linked to white spotting, i.e., saddleback, only in geese with European domestic goose ancestry. Hence, for taiga bean geese and geese with F‐haplotypes (either domestic or wild), we were unable to rule out wild status, as these geese (samples JH24, JH1 and JH40) did not carry the sex‐linked dilution allele (Table 1). These specimens were the oldest in our dataset (4th–13th centuries CE). During the High Medieval period (11th–14th centuries CE), one goose with a F‐haplotype (JH35; domestic or wild) had a T/T genotype upstream of the *EDNRB2* and was wild type (no deletion) in exon 4 of the *MLANA* gene. This goose was of the wild type solid colour, and hence, we were unable to determine whether it was a wild greylag or a grey domestic goose (site 11 in Figure 2). The same site also harboured a domestic goose with G/G genotype upstream of the *EDNRB2* and wild type genotype (no deletion) in exon 4 of the *MLANA* gene (JH34). Due to the strong linkage (Olli et al. 2026), we believe this individual was a saddleback (site 11 in Figure 2). A similar genotype, and thus a saddleback domestic goose, was observed at another site within the same time period (JH28, site 15 in Figure 2).

**FIGURE 2:**
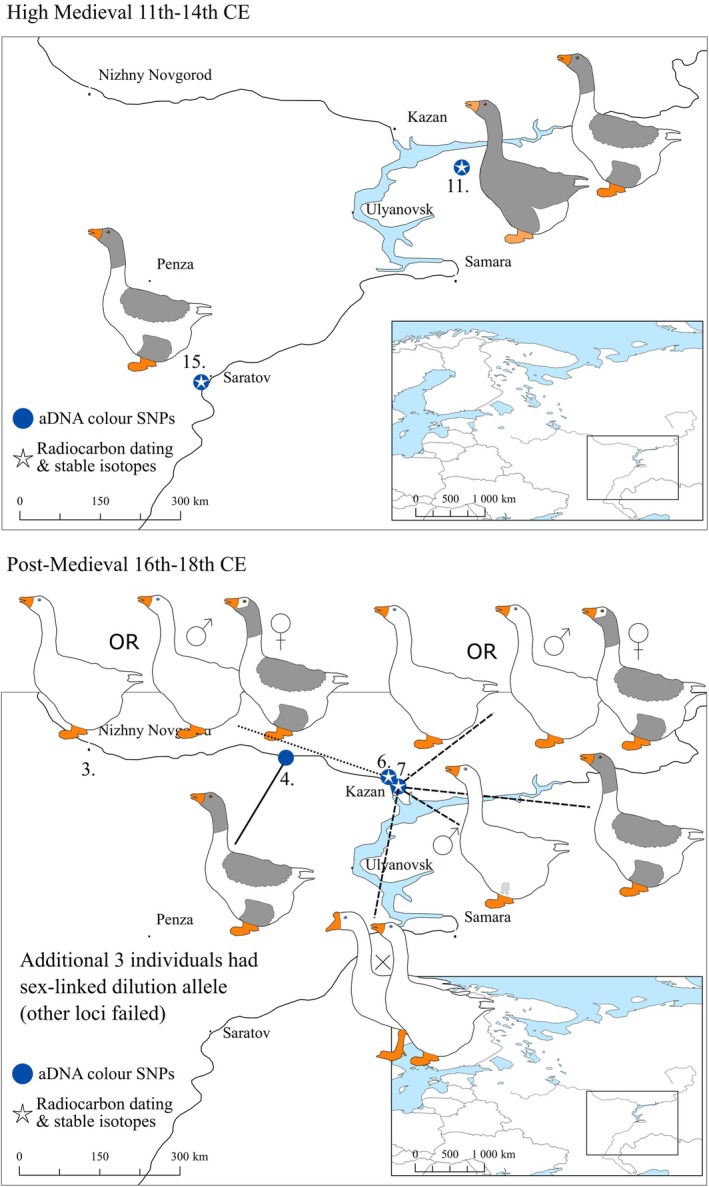
Inferred plumage colours of archaeological geese (*n* = 12; *Anser anser* ‐ancestry) based on genotyping of three loci in High‐Medieval (11th–14th century CE) and Post‐Medieval (15th–18th century CE). Plumage types included wild type solid colour, saddleback, nearly white with a variable amount of grey spotting, and European white or autosexing of the type ‘almost white male and saddleback female’. This type of autosexing is indistinguishable from white in the studied loci, as another unknown, and probably sex‐linked, locus allows the underlying saddleback pattern to be visible in females. Additionally, one individual is white due to a combination of Chinese domestic goose (*A. cygnoid*) white and European white/autosexing ‘almost white male and saddleback female’, indicated with an X between Chinese and European domestic geese. The site numbering is the same as in Figure 1.

Two probable saddleback individuals (JH4 and JH61) were also observed from the Post‐Medieval period (16th–18th centuries CE; sites 4 and 7 in Figure 2). Eight individuals had the sex‐linked dilution allele, i.e., a 1‐bp deletion in exon 4 of the *MLANA* gene, and could be confidently identified as domestic geese. Sex‐linked dilution without spotting dilutes females to light grey, often with white around the eyes and on the forehead (‘spectacles’), while males are diluted to almost white with some grey feathers on the rump, wings, and tail. However, for three individuals (JH8, JH13 and JH65, Table 1), this was the only successfully amplified locus, and we could not determine the phenotype. Additionally, for one goose (JH62), we obtained a genotype A/A upstream of *EDNRB2*, which could be due to post‐mortem or polymerase errors, but this locus did not amplify again despite repeated attempts. One goose was heterozygous for the sex‐linked dilution and thus a male, with a G/G genotype upstream of *EDNRB2* (JH2, Table 1). This combination produces white males with varying amounts of grey spots, which can be so small that the males are mistaken for white (site 7 in Figure 2; Olli et al. 2026). Two geese were homozygous or hemizygous for the sex‐linked dilution and had G/G genotype upstream of *EDNRB2* (JH18 and JH59; Table 1). This genotype is observed in both white and autosexing geese of the type ‘almost white male and saddleback female’ (sites 6 and 7 in Figure 2; Olli et al. 2026), thus the phenotype remained uncertain.

Further, one goose (JH17) also had the white or autosexing ‘almost white male and saddleback female’ genotype, but was also homozygous for the Chinese domestic goose white, i.e., the 14‐bp insertion in exon 3 of the *EDNRB2* gene (site 7 in Figure 2). Due to the presence of multiple whitening alleles, we believe this goose was white. The presence of both European and Chinese domestic goose alleles indicates that this individual was a hybrid between the two domestic goose types. A crossbred African goose has been reported in Europe in the 18th century (Kear 1990), but otherwise, the history of crossbreeding is unknown. Here, we show that this one individual had a crossbred background already in the 16th–17th century CE. How common interbreeding between the two domestic varieties was in the past remains an open question, as this sole individual could be an exception. On the other hand, the markers used here could not distinguish whether the geese were interbred with brown, aka grey Chinese domestic geese. Many current domestic goose breeds have Chinese domestic goose ancestry (> 10%) based on a genome‐wide study (Heikkinen et al. 2020), with another genomic study proving considerable Chinese domestic goose ancestry in the Rhine and Sebastopol goose breeds (Chen et al. 2023). Further genomic work with aDNA is warranted to study the extent of crossbreeding practices in the past.

We cannot rule out the effect of other colour loci on the phenotype of the geese. European domestic geese are known to have also blue (heterozygous for a blue allele), silver/lavender (homozygous for the blue allele), and buff phenotypes, as well as lilac (buff and heterozygous for a blue allele) and cream (buff and homozygous for the blue allele) phenotypes (Olli et al. 2026 ). However, the genetic basis for these traits is unknown (Olli et al. 2026). For example, a saddleback individual could be a buff‐coloured saddleback. Additionally, the extent of the saddleback pattern is variable, which could be caused by another locus (Ashton and Ashton 2022, 2023). There also appears to be variation in the “whiteness” of the white geese, with some white breeds producing goslings with lighter plumage (Ashton and Ashton 2023). The true phenotypes of the geese are probably not as simplistic as presented in this study, with multiple loci affecting the plumage colour or its variation. In our opinion, however, geese harbouring especially the sex‐linked dilution allele are likely domesticated, due to the perfect association of this trait with the *MLANA* mutation (Olli et al. 2026). Additionally, the co‐occurrence of the sex‐linked dilution and the Chinese domestic geese‐specific 14‐bp insertion in the *EDNRB2* gene in the same individual is a sign of crossbreeding between the two domestic goose forms.

## Author Contributions

**Johanna Honka:** conceptualization, investigation, writing – original draft, writing – review and editing, visualization, formal analysis. **Suvi Olli:** investigation, writing – review and editing, formal analysis. **Jouni Aspi:** conceptualization, writing – review and editing. **Laura Kvist:** conceptualization, writing – review and editing, funding acquisition. **Igor V. Askeyev:** conceptualization, writing – review and editing. **Arthur O. Askeyev:** conceptualization, writing – review and editing. **Dilyara N. Shaymuratova:** conceptualization, writing – review and editing. **Oleg V. Askeyev:** conceptualization, writing – review and editing.

## Funding

This work was supported by the Biodiverse Anthropocenes Research Programme of the University of Oulu, PROFI6, supported by the Research Council of Finland. Research Council of Finland, 370831.

## Conflicts of Interest

The authors declare no conflicts of interest.

## Supporting information


**Table S1:** Archaeological goose samples from Russia, analysed for colour loci in this study (*n* = 51). The mitochondrial DNA (mtDNA) haplotype.

## Data Availability

The data that support the findings of this study are available on request from the corresponding author. The sequence data is not publicly available due to sequence length restriction in GenBank.
