# Supplementary material for: Ancient Plumage Colour Genetics Reveal Goose Domestication and Hybridization
Source: Anim Genet. 2026 Jul 12;57(4):e70165. doi: 10.1002/age.70165 (PMC13357975; doi:10.1002/age.70165)
Supplement: Supplementary file 1 — Table S1: Archaeological goose samples from Russia, analysed for colour loci in this study (n = 51). The mitochondrial DNA (mtDNA) haplotype. [file AGE-57-0-s001.pdf]

Supplementary material

Table S1. Archaeological goose samples from Russia, analysed for colour loci in this study (*n* = 51). The mitochondrial DNA (mtDNA) haplotype was determined in Honka et al. (2018) using a 204 bp fragment. Haplotype names were determined based on published goose sequences, with forward slashes between haplotype names denoting haplotypes that were identical in the studied 204 bp fragment, but which differed in the longer control region sequence (1249 bp). Archaeological information (species based on bone morphology, studied bone fragment, archaeological dating, radiocarbon age, archaeological site and location) is also indicated, with the radiocarbon age of selected samples determined in Honka et al. (2026). The genotypes in the three analysed colour loci are also given with *MLANA* = *melan-A* gene and *EDNRB2* = *endothelin receptor B-like*, as well the predicted plumage phenotype based on these three loci as in Olli et al. (2026). Due to sex-linkage in the *MLANA* gene, it was impossible to differentiate homozygous males and hemizygous females, and thus we only show one allele (unless a heterozygous male) with C (cytosine) = no dilution, i.e., wild-type and -1 bp = sex-linked dilution. N/A = no PCR product was observed in agarose gel, and further genotyping was not attempted with other loci. In the locus upstream of the *EDNRB2* gene T (thymine) = linked to solid colour, i.e., wild type in the European domestic goose and G (guanine) = linked to spotting in the European domestic goose. In exon 3 of the *EDNRB2,* - = no insertion, i.e. wild-type and 14-bp insertion = Chinese domestic goose white. Genotypes in parentheses indicate that the genotyping was successful only once out of two or three attempts.

**Sample name**	**Haplo-type**	**Species based on mtDNA**	**Species based on bone morpho-logy**	**Studied bone fragment**	**Archaeo-logical dating**	**Time period**	**Radio-carbon age Cal CE 95.4%**	**Archaeo-logical site**	**Location of site**	**exon 4 in *MLANA***	**up-stream of *EDNRB2***	**14-bp insertion in exon 3 of *EDNRB2***	**Inferred plumage colour**
JH24	Fa5	Taiga bean goose	Domestic goose	ulna/ humerus	4th -8th centuries CE	Early Medieval		Tetyushskoe II hillfort	Tatarstan Republic	C	(T/G) §	-/-	? taiga bean goose
JH25	Fa5	Taiga bean goose	Domestic goose	humerus	4th -8th centuries CE	Early Medieval		Tetyushskoe II hillfort	Tatarstan Republic	N/A			
JH1	F11	Domestic goose/ Wild greylag goose	Domestic goose	tibiotarsus	4th -8th centuries CE	Early Medieval	600-675	Tetyushskoe II hillfort	Tatarstan Republic	C(C/T*)	G/G	-/-	? domestic /wild

JH23	F11	Domestic goose/ Wild greylag goose	Domestic goose/ Wild greylag goose	furcula	5th -7th centuries CE	Early Medieval		Imenkov hillfort	Tatarstan Republic	N/A			
JH39	FAB1	Taiga bean goose	Domestic goose	tibiotarsus	9th - 10th centuries CE	Early Medieval		Staraya Ladoga	Leningrad Region	N/A			
JH40	Fa3	Taiga bean goose	Domestic goose	femur	9th - 10th centuries CE	Early Medieval	893-1026	Staraya Ladoga	Leningrad Region	C	G/G	-/-	? taiga bean goose
JH37	F6	Domestic goose/ Wild greylag goose	Domestic goose/ Wild greylag goose	humerus	11th -12th centuries CE	High Medieval	1167-1267	Ostolopovsk oe settlement	Tatarstan Republic	N/A			
JH38	D3/D7	Domestic goose	Domestic goose/ Wild greylag goose	tarsome-tatarsus	11th -12th centuries CE	High Medieval	1030-1160	Ostolopovsk oe settlement	Tatarstan Republic	N/A			
JH29	Fa6	Taiga bean goose	Domestic goose/ Wild greylag goose	femur	11th -13th centuries CE	High Medieval	1044-1220	Bilyarsk (Defensive moat location-peripherals of settlement)	Tatarstan Republic	N/A			
JH30	D3/D7	Domestic goose	Domestic goose	humerus	12th -13th centuries CE	High Medieval		Bilyarsk (Palace lord location - downtown)	Tatarstan Republic	N/A			
JH31	D3/D7	Domestic goose	Domestic goose	tibiotarsus	12th -13th centuries CE	High Medieval		Bilyarsk (Palace lord location - downtown)	Tatarstan Republic	N/A			
JH56	D4/D5	Domestic goose	Domestic goose/ Wild greylag goose	ulna	12th -13th centuries CE	High Medieval		Elabuga hillfort	Tatarstan Republic	N/A			

JH28	D4/D5	Domestic goose	Domestic goose	sternum	13th -14th centuries CE	High Medieval	1275-1389 Marine correc-tion: 1486-1888	Bagaevskoe settlement	Saratov Region	C	G/G	-/-	Saddle-back
JH47	D3/D7	Domestic goose	Domestic goose/ Wild greylag goose	humerus	13th -14th centuries CE	High Medieval	1278-1390	Nizhny Novgorod Kremlin	Nizhny Novgorod Region	N/A			
JH48	D3/D7	Domestic goose	Domestic goose/ Wild greylag goose	tibiotarsus	13th -14th centuries CE	High Medieval		Nizhny Novgorod Kremlin	Nizhny Novgorod Region	N/A			
JH49	D4/D5	Domestic goose	Domestic goose/ Wild greylag goose	tarsome-tatarsus	13th -14th centuries CE	High Medieval		Nizhny Novgorod Kremlin	Nizhny Novgorod Region	N/A			
JH50	D4/D5	Domestic goose	Domestic goose/ Wild greylag goose	tibiotarsus	13th -14th centuries CE	High Medieval		Bulgar	Tatarstan Republic	N/A			
JH51	F6	Domestic goose/ Wild greylag goose	Domestic goose/ Wild greylag goose	tibiotarsus	13th -14th centuries CE	High Medieval		Bulgar	Tatarstan Republic	N/A			
JH52	Fa3	Taiga bean goose	Domestic goose/ Wild greylag goose	ulna	13th -14th centuries CE	High Medieval	899-1120	Bulgar	Tatarstan Republic	(C)		(-/-)	
JH53	D4/D5	Domestic goose	Domestic goose /Wild greylag goose	tarsome-tatarsus	13th -14th centuries CE	High Medieval		Bulgar	Tatarstan Republic	N/A			

JH32	D3/D7	Domestic goose	Domestic goose	humerus	15th century CE	Late Medieval		Toretskoe settlement	Tatarstan Republic	N/A			
JH33	D3/D7	Domestic goose	Domestic goose	humerus	15th century CE	Late Medieval		Toretskoe settlement	Tatarstan Republic	(C)			
JH34	D3/D7	Domestic goose	Domestic goose	humerus	15th century CE	Late Medieval	1274-1388	Toretskoe settlement	Tatarstan Republic	(C)	(G/G)	(-/-)	(Saddle-back)
JH35	F6	Domestic goose/ Wild greylag goose	Domestic goose	humerus	15th century CE	Late Medieval	1283-1395	Toretskoe settlement	Tatarstan Republic	(C)	(T/T)		(Wild type)
JH16	D4/D5	Domestic goose	Domestic goose	radius	16th -17th centuries CE	Early Post-Medieval		Kazan city (territory of Kazan University)	Tatarstan Republic	N/A			
JH17	D4/D5	Domestic goose	Domestic goose	humerus	16th -17th centuries CE	Early Post-Medieval		Kazan city (territory of Kazan University)	Tatarstan Republic	-1 bp	G/G	(14 bp insertion/ 14 bp insertion) §	White and Chinese domestic goose white
JH18	D4/D5	Domestic goose	Domestic goose	humerus	16th -17th centuries CE	Early Post-Medieval		Kazan city (territory of Kazan University)	Tatarstan Republic	-1 bp	G/G	-/-	White or auto-sexing white male/ saddle-back female
JH2	D4/D5	Domestic goose	Domestic goose	humerus	16th -17th centuries CE	Early Post-Medieval		Kazan city (territory of Kazan University)	Tatarstan Republic	C/-1 bp	G/G	-/-	Almost white male with grey spots

JH3	D4/D5	Domestic goose	Domestic goose	humerus	16th -17th centuries CE	Early Post-Medieval	1527-modern	Kazan city (territory of Kazan University)	Tatarstan Republic	N/A			
JH4	D4/D5	Domestic goose	Domestic goose	humerus	16th -17th centuries CE	Early Post-Medieval		Kazan city (territory of Kazan University)	Tatarstan Republic	(C)	(G/G)	(-/-)	(Saddle-back)
JH5	D4/D5	Domestic goose	Domestic goose	humerus	16th -17th centuries CE	Early Post-Medieval		Kazan city (territory of Kazan University)	Tatarstan Republic	N/A			
JH6	D3/D7	Domestic goose	Domestic goose	coracoi-deum	16th -17th centuries CE	Early Post-Medieval		Kazan city (territory of Kazan University)	Tatarstan Republic	N/A			
JH7	D4/D5	Domestic goose (possible duplicate of the humerus in this site)	Domestic goose	coracoi-deum	16th -17th centuries CE	Early Post-Medieval		Kazan city (territory of Kazan University)	Tatarstan Republic	N/A			
JH8	D3/D7	Domestic goose	Domestic goose	coracoi-deum	16th -17th centuries CE	Early Post-Medieval		Kazan city (territory of Kazan University)	Tatarstan Republic	(-1 bp)			
JH10	D3/D7	Domestic goose	Domestic goose	femur	16th -17th centuries CE	Early Post-Medieval		Kazan city (territory of Kazan University)	Tatarstan Republic	N/A			
JH11	D4/D5	Domestic goose (possible duplicate of the humerus in this site)	Domestic goose	femur	16th -17th centuries CE	Early Post-Medieval		Kazan city (territory of Kazan University)	Tatarstan Republic	N/A			

JH12	D4/D5	Domestic goose (possible duplicate of the humerus in this site)	Domestic goose	tibiotarsus	16th -17th centuries CE	Early Post-Medieval		Kazan city (territory of Kazan University)	Tatarstan Republic	N/A			
JH13	D4/D5	Domestic goose (possible duplicate of the humerus in this site)	Domestic goose	tarsome-tatarsus	16th -17th centuries CE	Early Post-Medieval		Kazan city (territory of Kazan University)	Tatarstan Republic	-1 bp			
JH14	D4/D5	Domestic goose (possible duplicate of the humerus in this site)	Domestic goose	carpometa-carpus	16th -17th centuries CE	Early Post-Medieval		Kazan city (territory of Kazan University)	Tatarstan Republic	N/A			
JH61	D3/D7	Domestic goose	Domestic goose	femur	16th -18th centuries CE	Early Post-Medieval		Cheboksary city	Chuvash Republic	C	G/G	-/-	Saddle-back
JH62	D3/D7	Domestic goose	Domestic goose	humerus	16th -18th centuries CE	Early Post-Medieval		Cheboksary city	Chuvash Republic	-1 bp	(A/A)*§	-/-	
JH64	D3/D7	Domestic goose	Domestic goose	tibiotarsus	16th -18th centuries CE	Early Post-Medieval		Cheboksary city	Chuvash Republic	N/A			
JH45	D4/D5	Domestic goose	Domestic goose/ Wild greylag goose	tibiotarsus	first half of 16th century CE	Early Post-Medieval	1457-1635	Pskov city (New Torg location)	Pskov Region	N/A			
JH46	D4/D5	Domestic goose	Domestic goose/ Wild greylag goose	tibiotarsus	first half of 16th century CE	Early Post-Medieval		Pskov city (New Torg location)	Pskov Region	N/A			
JH54	D3/D7	Domestic goose	Domestic goose	humerus	17th – 18th centuries CE	Early Post-Medieval		Elabuga hillfort	Tatarstan Republic	N/A			

JH55	D3/D7	Domestic goose	Domestic goose	tibiotarsus	17th – 18th centuries CE	Early Post-Medieval		Elabuga hillfort	Tatarstan Republic	N/A			
JH65	D4/D5	Domestic goose	Domestic goose	humerus	17th century CE	Early Post-Medieval		Sviyazhsk	Tatarstan Republic	(-1 bp)			
JH66	D4/D5	Domestic goose	Domestic goose	tibiotarsus	17th century CE	Early Post-Medieval		Sviyazhsk	Tatarstan Republic	N/A			
JH58	F6	Domestic goose/ Wild greylag goose	Domestic goose	furcula	18th century CE	Late Post-Medieval		Kazan Kremlin	Tatarstan Republic	N/A			
JH59	D4/D5	Domestic goose	Domestic goose	humerus	18th century CE	Late Post-Medieval		Kazan Kremlin	Tatarstan Republic	(-1 bp)	(G/G)	(-/-)	(White or auto-sexing white male/ saddle-back female)
JH60	D4/D5	Domestic goose	Domestic goose	humerus	18th century CE	Late Post-Medieval	1488-1643	Kazan Kremlin	Tatarstan Republic	N/A			

* Postmortem changes

§ The sample was attempted thrice, but was successfully amplified only once

References:

Honka, J., Heino, M. T., Kvist, L., Askeyev, I. V., Shaymuratova, D. N., Askeyev, O. V., Askeyev, A. O., Heikkinen, M. E., Searle, J. B., & Aspi, J. (2018). Over a thousand years of evolutionary history of domestic geese from Russian archaeological sites, analysed using ancient DNA. *Genes*, *9*(7). https://doi.org/10.3390/genes9070367

Honka, J., Massé, C., Askeyev, A. O., Askeyev, I. V., Askeyev, O. V., Aspi, J. Shaymuratova, D. N., & Kvist, L. (2026). Stable carbon and nitrogen isotope analysis explores diverse domestic goose management practices in Medieval and Postmedieval Russia. *International Journal of Osteoarchaeology*, *36*(3), 537–553. https://doi.org/10.1002/oa.70097

Olli, S., Ahola, V., Heikkinen, M. E., & Honka, J. (2026). Sex-linked dilution colour in the European domestic goose indicated to be a 1-bp deletion in the *Melan-A* gene. *Animal Genetics, 57*(4), e70161. https://doi.org/10.1002/age.70161.
